# Mycotoxin Detection in Maize, Commercial Feed, and Raw Dairy Milk Samples from Assiut City, Egypt

**DOI:** 10.3390/vetsci6020057

**Published:** 2019-06-18

**Authors:** Mohamed F. Abdallah, Gözde Girgin, Terken Baydar

**Affiliations:** 1 Department of Forensic Medicine and Toxicology, Faculty of Veterinary Medicine, Assiut University, Assiut 71515, Egypt; 2 Department of Pharmaceutical Toxicology, Faculty of Pharmacy, Hacettepe University, Sihhiye, 90-06100 Ankara, Turkey; ggirgin@hacettepe.edu.tr (G.G.); tbaydar@hacettepe.edu.tr (T.B.)

**Keywords:** mycotoxins, maize, raw milk, aflatoxins, ochratoxin a, zearalenone, HPLC, Egypt

## Abstract

This survey was conducted to investigate the contamination by multiple mycotoxins, aflatoxins (AFB_1_, AFB_2_, AFG_1_, and AFG_2_), ochratoxin A (OTA), and zearalenone (ZEA) in 61 samples of maize and 17 commercial animal feed samples, and of aflatoxin M_1_ (AFM_1_) in raw dairy milk samples (n = 20) collected from Assiut City in Upper Egypt. Multi-mycotoxin immunoaffinity columns were used for samples cleanup and mycotoxin purification. An HPLC–FLD system with an on-line post-column photochemical derivatization was used for the detection of the target toxins. AFB_1_ was detected in both maize (n = 15) and feed (n = 8), with only one maize sample presenting a concentration above the maximum permissible level set by the Egyptian authorities. AFB_2_ was observed in six maize samples and in one feed sample, with a maximum value of 0.5 μg/kg. ZEA was detected only in feed samples (n = 4), with a maximum value of 3.5 μg/kg, while OTA, AFG_1_, and AFG_2_ were under the limits of detection. For milk, all the analyzed samples (100%) were contaminated with AFM_1_, and 14 samples (70%) presented concentrations above the maximum permissible level in the European Union (EU) (0.05 μg/kg). The concentrations ranged from 0.02 μg/kg to 0.19 μg/kg, except that of one sample, which was under the limit of quantification. The contamination rates in maize and animal feeds are not alarming. In contrast, the consumption of dairy milk samples in Assiut City may pose public health hazards, as AFM_1_ levels were found to exceed the international permissible limits. Further surveys are highly recommended in order to establish a database for mycotoxin occurrence in Egypt to minimize the possible health risks in animals and humans.

## 1. Introduction

Mycotoxins are secondary metabolites produced by different species of fungi such as *Aspergillus*, *Penicillium*, *Fusarium* and *Alternaria* [1,2]. These fungi are very diverse and can grow under a wide array of climatic conditions [3]. The diversity of mycotoxin structures induces various toxic effects in mammals, poultry, and fish. The health impacts depend on several factors such as species, age, gender, health, and physiological state of the exposed organism [4]. Some of these effects may be carcinogenic, mutagenic, teratogenic, nephrotoxic, hepatotoxic, estrogenic, hemorrhagic, neurotoxic, immunotoxic, and dermotoxic [3,5]. In addition, more than one toxic effect may occur in the case of multiple mycotoxins co-exposure, owing to their possible synergistic and/or additive effects, or in the presence of other types of natural or synthetic contaminants.

Several outbreaks were reported in humans and animals after consumption of mycotoxin-contaminated foods. Because of their serious impacts on health and worldwide economy, more than 100 countries adopted specific maximum mycotoxin limits in 2003. However, still most of the African countries have no specific mycotoxin regulations [6]. The absence of these regulations is mainly related to insufficient data on the occurrence of mycotoxins. Among these countries, Egypt regulates only the maximum permissible level of total AFs and AFB_1_ concentrations, 20 μg/kg and 10 μg/kg, respectively, for both animal feed and maize [7]. Moreover, without reliable data on mycotoxins occurrence, performing assessment of exposure may not be feasible.

On the other hand, several methods have been developed in order to simultaneously detect mycotoxins in different matrices, especially maize and feeds, using HPLC coupled with a fluorescence detector (FLD) [8,9]. The literature shows that most of the previous surveys conducted in Egypt have focused on a single or a few mycotoxins in maize and/or feed using screening methods for qualitative detection. For example, thin layer chromatography (TLC) was used for the detection of total aflatoxins (AFs), aflatoxin B_1_ (AFB_1_), and zearalenone (ZEA) in maize by El-Tahan et al., 2000 [10], El-Gohary, 1995 [11], and Abd Alla, 1997 [12], respectively. Total AFs in feedstuffs [13] and fumonisins in maize [14] from Assiut City were surveyed using ELISA. Ochratoxin A (OTA) was rarely investigated in Egypt. Because of the absence of sufficient and up-to-date surveys on mycotoxin contamination in Egypt, especially in the upper (south) part of the country, the aim of the present work was to survey the natural occurrence of three classes of the most researched mycotoxins worldwide, i.e., AFs, OTA, and ZEA in maize and commercial feed marketed in Assiut, Egypt. In addition, AFM_1_ in raw dairy milk samples from the same area was also analyzed.

## 2. Materials and Methods

### 2.1. Sample Collection

All samples, maize (n = 61) and commercial animal feed (n = 17), were randomly collected between summer 2014 and winter 2015 (250 g each) from generic local markets as well as from farms located across Assiut Governorate in Upper Egypt. In addition, 20 raw cow milk samples (250 mL each) intended for human consumption were purchased from different local markets in the same area during the summer season of 2014. The identities of markets, local vendors of raw milk, and commercial manufacturers of feeds and maize cannot be disclosed because of the confidential nature of this information. The samples were transported in polystyrene boxes with a cooling gel (pre-frozen to −20 °C) to Ankara, Turkey, for extraction and mycotoxin quantification using HPLC–FLD. All samples were stored at −20 °C until the time of analysis. 

### 2.2. Sample Preparation and Extraction 

#### 2.2.1. Maize and Animal Feed Samples

Each sample (250 g) of maize and feed was ground by using a laboratory blender (Osterizer^®^ Blender, Waring commercial, Cheadle, UK) at high speed for 1–2 min. Sample extraction was done according to the instructions of the manufacturer and as published by Göbel and Lusky (2004) [15]. Each sample (25 g) was mixed with 5 g of sodium chloride in 100 mL of acetonitrile and purified water (80:20, *v*/*v*) and blended for 2 min at high speed. The mixture was filtered by fluted filter paper (Vicam, Nixa, MO, USA), and 10 mL of the extract was diluted with 40 mL of phosphate-buffered saline (PBS) (pH 7.4) containing 0.01% Tween 20. The mixture was vortexed, and a second filtration was performed using a microfiber filter paper (Vicam, Nixa, MO, USA). An aliquot of 20 mL of the filtrate was passed through a multiple-mycotoxin immunoaffinity columns (AOZ IAC) purchased from Vicam (Nixa, MO, USA), by a syringe barrel, at a flow rate of 1–2 drops/second. The column were washed with 10 mL PBS containing 0.01% Tween 20 followed by 10 mL of purified water. The IAC columns were dried gently by passing air for 2–3 s and then washed with purified water. The target toxins were eluted from the IAC columns by passing 1.5 mL of methanol (Sigma, Darmstadt, Germany) and 1.5 mL 0.1% acetic acid (Sigma, Germany) and collected in one glass tube. After vortexing, the final mixture was transferred into HPLC injection vials and subjected to analysis.

#### 2.2.2. Dairy Milk Samples Extraction

AFM_1_ extraction and detection was done according to the protocol published by Dragacci et al. (2001) [16]. Each raw milk sample (250 mL) was centrifuged at 3190 RCF (Hettich Universal Rotina 420 R, Germany) for 15 min at room temperature (RT) to get rid of the fatty layer. Afterwards, the samples were filtered through fluted filter paper (Vicam, Nixa, MO, USA) and microfiber filter paper (Vicam, Nixa, MO, USA). The skimmed milk samples (each 50 mL) were loaded by a syringe barrel Afla M_1_ immunoaffinity columns (Vicam, Nixa, MO, USA) at a rate of 1–2 drops/second. The columns were washed twice with 10 mL of purified water at a rate of 1–2 drops/second to get rid of impurities, and the toxin was eluted from the column by passing 1.25 mL of a solution of acetonitrile/methanol (3:2, *v*/*v*) at a rate of 1 drop for every 2–3 s, followed by an equal amount of purified water. The final solution was collected in a glass cuvette and transferred into HPLC injection vials for analysis.

#### 2.2.3. HPLC Parameters for Multi-Mycotoxin and AFM_1_

**Figure 1:**
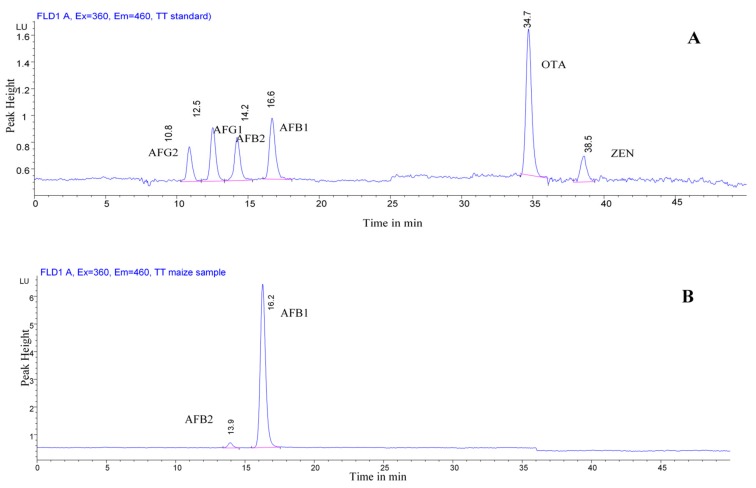
HPLC–fluorescence detector (FLD) chromatograms of a standard aflatoxin (AF) mixture (**A**) and a maize sample contaminated with AFB_1_ and AFB_2_) (**B**).

The chromatographic parameters for the analysis are described in Ofitserova et al. (2009) [9]. Chromatographic separation was performed by using an Agilent 1100 HPLC equipped with an ACE^®^ C18 column (25 cm × 4.6 mm, particle size 5 µm) and a C18 4 × 3 mm i.d. security guard cartridge (Aberdeen, Scotland). The HPLC device was coupled with a fluorescence detector for quantitative determination with on-line post column photochemical derivatization. The photochemical reactor (Vicam, Nixa, MO, USA) was used for aflatoxins (AFB_1_ and AFG_1_) to enhance the sensitivity and/or selectivity of the fluorescence detection response avoiding a decrease of sensitivity to AFB_2_ and AFG_2_, ZEA and OTA. The flow rate was set at 0.8 mL/min, and the column temperature at 40 °C. The injection volume was 100 µL, and the total running time was 50 min including 10 min for equilibration. Figure 1 shows a chromatogram of a standard mix solution containing the six analytes.

**Figure 2:**
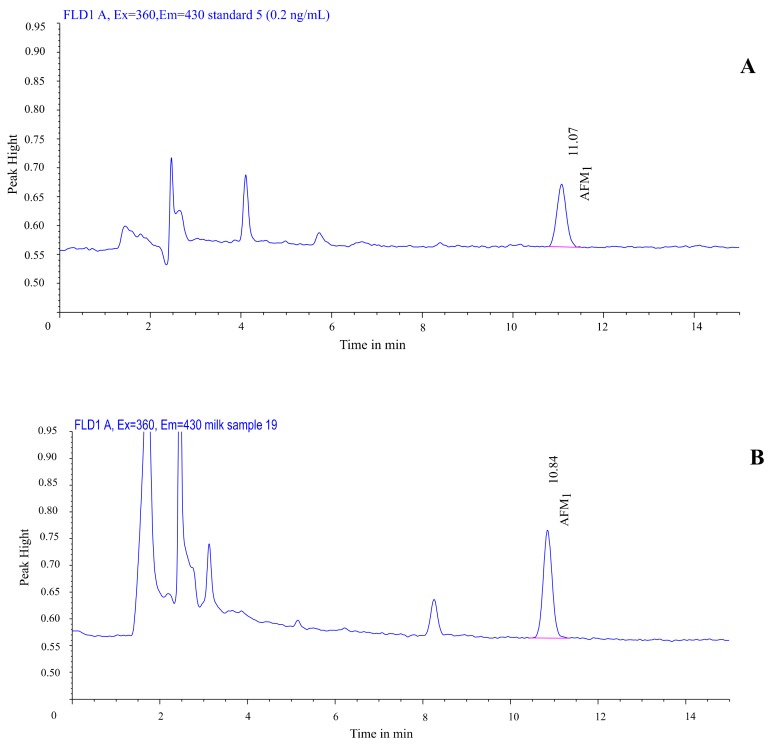
HPLC–FLD chromatograms of a standard solution (**A**) and a milk sample contaminated with aflatoxin M_1_ (**B**).

For AFM_1_, the chromatographic separation was performed at 25 ± 1 °C using HPLC equipped with a Hichram^®^ ODS2 column (250 mm × 4.6 mm i.d., 5 μm particle size) and C18 (4 × 3 mm i.d.) security guard cartridge. The mobile phase consisted of methanol/water/acetonitrile (22:62:16; *v*/*v*/*v*) in isocratic elution. The fluorescence detector was adjusted with excitation and emission wavelengths of 360 nm and 430 nm, respectively. The injection volume was 100 µL. The flow rate was 1 mL/ min, the retention time was 10.7 ± 0.2 min, and the running time was 15 min. An HPLC chromatogram for the AFM_1_ (0.2 µg/kg) standard solution is shown in Figure 2.

The sensitivity of the method was tested by examining both limit of detection (LOD) and limit of quantification (LOQ). LOD was calculated based on signal to noise (S/N = 3) while LOQ was (S/N = 10). LOD values were 0.92 μg/kg for ZEA, 0.02 μg/kg for OTA and varied from 0.04 to 0.12 μg/kg for aflatoxins. LOQ values were 2.8 μg/kg for ZEA, 0.06 μg/kg for OTA, and from 0.12 to 0.39 μg/kg for aflatoxins. The efficacy of the analytical method was determined by estimation of apparent recovery. The mean apparent recoveries ranged from 81 to 110% for different concentrations (cut of the calibration curve) of the target mycotoxins (Table 1 and Table 2) in two spiked samples of maize and feed. For AFM_1_, LOD and LOQ values were 0.008 μg/kg and 0.02 μg/kg, respectively. The recovery was performed once and was 100%.

## 3. Results and Discussion

### 3.1. Occurrence of Mycotoxins in Maize and Animal Feed Samples

During the past few years, the simultaneous determination of several toxins in one analytical run was developed with the aim of reducing time and cost of the analysis and to get a better evidence of multi-mycotoxin occurrence in agriculture commodities [9,15,17]. HPLC has proved to be a powerful tool for mycotoxin detection and quantification. Cereals, especially maize and its products, are important agricultural commodities for feeding humans and animals. However, they can also pose risks to health if they are contaminated with mycotoxins. In fact, it is estimated that up to 100% of crops are contaminated by one mycotoxin at a low level [18].

In both feed and maize samples (n = 78), the most prevalent mycotoxin was AFB_1_. In total, 23 feed and maize samples were contaminated with AFB_1_, 7 samples with AFB_2_, and 4 samples with ZEA. Regarding feeds, 47% (n = 8) of the samples were contaminated with AFB_1_, while 23% of maize samples (n = 15) were contaminated with for AFB_1_. Also, the maximum level of AFB_1_ in feed was much lower than that detected in maize. ZEA was detected only in feed samples, while OTA, AFG_1_, and AFG_2_ were not detected in either matrix. The prevalence, mean, and median values are presented in Table 1 and Table 2.

AFs are mainly produced by several species of *Aspergillus* section *Flavi* [3,4]. The four types of AFs (B_1_, B_2_, G_1_, and G_2_) are ubiquitous in food and feed stuffs and contaminate many commodities including peanuts, rice, maize, cottonseed, almonds, spices, sugarcane, palm dates [19], and figs [17,20,21,22]. AFs are by far the most intensively researched toxins due to their potent acute toxicity and chronic hepatocarcinogenic effects in various susceptible animal species. Although the liver is the primary target organ, under certain conditions, lung, kidney, and colon may be also affected [1,4,23]. The International Agency for Research on Cancer (IARC) has classified aflatoxin B_1_ and naturally occurring mixtures of aflatoxins as human carcinogens (group 1) [24]. The highest incidence of hepatocellular carcinoma occurs in areas where people are frequently exposed to contaminated food and have a high rate of infection with hepatitis, such as Eastern and South Eastern Asia and Middle and Western Africa [3,21].

Our results confirmed the presence of AFB_1_ in maize from Egypt at levels higher than the national and international limits. This data are also in agreement with the previous reported levels in maize from Cairo (19.2 µg/kg) [25] and from Assiut (21.8 µg/kg) [26]. AFB_1_ level in feed was lower than those reported in feedstuff from different regions of Egypt, where up to 400 µg/kg were detected, [27] and from Assiut, where 60 µg/kg were detected [13].

OTA is the most commonly encountered and toxic metabolite of the ochratoxin group. The metabolite is a fluorescent secondary metabolite produced by two genera of fungi, *Aspergillus* and *Penicillium* [4]. The toxin is a frequent natural contaminant in food, including various cereal products, coffee, spices, bean, as well as dried fruits, grapes, and grape-based products such as wine. Moreover, edible animal tissues and milk were reported to be contaminated with OTA [5,28]. The main target organ involved in OTA toxicity is the kidney, and the toxin may have some carcinogenic, genotoxic, immunotoxic, and potent nephrotoxic effects. In the Balkan countries, OTA has been linked to a high incidence of urinary tract carcinomas, a condition known as Balkan Endemic Nephropathy [1,3]. OTA has been classified by IARC as a possible carcinogen to humans (group 2B). OTA is not a frequent food and feed contaminant in Egypt, as confirmed by a recent survey from Upper Egypt, where the authors screened for a wide range of fungal metabolites in animal feed and maize but could not detect OTA [17].

ZEA is a mycotoxin with hyperestrogenic effects produced by *Fusaria*, mainly *Fusaria graminearum*, *Fusaria culmorum*, and *Fusaria cerealis*. The toxin is a frequent contaminant of several cereal crops worldwide, especially maize [5]. ZEA resembles 17 β-estradiol and is classified as a non-steroidal oestrogenic mycotoxin, which causes mainly reproductive problems in domestic farm animals, especially swine. The toxin is also suspected to cause precocious puberty in humans. ZEA is considered not classifiable with regard to its carcinogenicity in humans (group 3) [24]. The level of ZEA detected in the present study was much lower than those detected in feed (791 μg/kg) and maize (184 μg/kg) from upper Egypt [17], which reached the maximum values; however, the measured level was higher than those reported for maize in Cairo (2.15 μg/kg) [29], in some Egyptian districts (45.2 μg/kg) [12], and in other surveys (3.5 μg/kg) [26,27]. Until now, there are no regulations for ZEA in any commodity in Egypt, and extensive surveys on different food and feed commodities should be conducted to gain more information about ZEA occurrence and the fungi that produce it in the Egyptian environment.

### 3.2. Occurrence of AFM_1_ in Dairy Raw Samples

The analysis using a reversed-phase liquid chromatography coupled with a fluorescence detector showed that all the examined milk samples were contaminated with AFM_1_. Among them, 14 samples (70%) presented concentrations above the maximum permissible level set by the EU (0.05 μg/kg) (EC, 2010). The detected concentrations in the samples ranged from 0.02 μg/kg to 0.19 μg/kg, except for only one sample whose concentration was under the LOQ. Figure 2 shows a chromatogram of a 0.2 µg/kg AFM_1_ standard and a naturally contaminated milk sample, containing 0.19 µg/kg AFM_1_.

Milk is a natural nutritious food for humans, especially for children. However, it also serves as a favorable environment for the growth of various microorganisms, and can be contaminated with other toxins through carry over from the dairy animals.

AFM_1_, the hydroxylated metabolic form of AFB_1_, is present in dairy milk of animals that ingested feedstuffs contaminated with AFB_1_ [28]. AFM_1_ is classified as a possible human carcinogen in Group 2B, as its carcinogenicity is 10 times less than that of the parent compound [24].

In general, the presence of AFM_1_ in milk depends exclusively on the absorbed concentrations of the precursor AFB_1_ in animal feeds. It has been estimated that around 5% of digested AFB_1_ is converted and excreted into the milk of dairy animals [1,5,30]. However, the amount of excreted AFM_1_ is subjected to individual variation in addition to a seasonal variation, according to the nature of feed in summer and winter. It has been reported that AFM_1_ can be detected after 12 h from the time of ingestion of AFB_1_-contaminated forages or animal concentrates by lactating dairy animals, and its level decreases gradually, becoming mostly undetectable within 72 h after the removal of AFB_1_-contaminated feeds. Also, the unsanitary conditions and/or contamination of the utensils used for milking or inside local markets for handling and boiling the raw milk might contribute to milk contamination with the toxin [31]. The toxin is not totally destroyed by pasteurization, autoclaving, and other food-processing procedures [32]. Yet, little is known about the natural occurrence of AFM_1_ in dairy products in Egypt. The determined levels of AFM_1_ observed in the present study (0.02–0.19 µg/kg) were lower than those reported previously from different districts of Egypt. For example, a very high level of contamination was reported (5–8 µg/kg) in 3 cow milk samples out of 15 samples collected from Cairo and Giza Governorates, using HPLC–FLD [31]. In another survey conducted in Ismailia Governorate, 0.01–0.2 µg/kg of AFM_1_ was detected in 50 samples of cow milk, using ELISA [32], and in a recent survey from Assiut Governorate, the AFM_1_ level detected was 0.09–0.5 µg/kg, again using ELISA [33].

On the other hand, Salem 2002 [13] reported up to 0.015 µg/kg of AFM_1_ in milk samples from Assiut Governorates using ELISA, and Ghareeb et al. (2013) [30] found the same prevalence of AFM_1_ in 48 raw milk samples (97.92%), with a concentration up to 0.11 µg/kg from Qena Governorates, using the same technique. The current results are generally in agreement with those of previous reports and reveal a serious public health problem for the Egyptian population living in Assiut Governorate, as it is exposed to AFM_1_ levels above the international limits. In most of the developing countries, including Egypt, raw milk is sold without or with a minimal traditional heat treatment that, in fact, does not affect mycotoxins and their metabolites, as they are in general heat-resistant. Because of the lack of data on the natural occurrence of AFs and other factors such as lack of laboratory analytical equipment, no official regulation has been established by the Egyptian authorities so far. Also, it should be kept in mind that the total daily aflatoxin and other mycotoxins intake from other types of food could be an additional important risk factor.

## 4. Conclusions

The present study presents the occurrence of multiple mycotoxins in maize and animal feed from Egypt using reversed-phase liquid chromatography coupled with a fluorescence detector. The results show that the mycotoxin contamination rates are not alarming for AFB_1_ in maize and animal feeds, according to the international standards. Also, the concentrations of AFB_1_ were below the authorized levels in these food samples. In contrast, the concentrations of AFM_1_ in most of the analyzed milk samples were above the international permissible level, and therefore, the consumption of dairy milk in Assiut City may pose a public health hazard. Further and permanent monitoring is highly recommended to establish a mycotoxin occurrence database. A stricter regulation for mycotoxin levels in food should be applied in Egypt in order to control mycotoxin and their producing fungi in animal feed, maize, and other food commodities. Furthermore, reducing the maximum level of the regulated mycotoxins to a level accepted by the international standards is important to ensure consumers’ safety and facilitate worldwide trade.

## Figures and Tables

**Table 1:** Overview of mycotoxin contents (µg/kg) in feed samples.

Mycotoxin	N (P)	Range (Min–Max)	Median	Mean	Recovery (%)
AFB_1_	8 (47%)	(0.1–5.9)	0.7	1.5	110
AFB_2_	1 (6%)	(0.5)	0.5	0.5	81
AFG_1_	n.d	n.d	n.d	n.d	79
AFG_2_	n.d	n.d	n.d	n.d	80
OTA	n.d	n.d	n.d	n.d	95
ZEA	4 (24%)	(1.0–11.9)	8.4	8.1	88.8

N/P, number of the contaminated samples over the percentage, OTA, ochratoxin A, ZEA, zearalenone, n.d, not determined. Median and mean values were calculated for the contaminated samples.

**Table 2:** Overview of mycotoxin contents (µg/kg) in maize samples.

Mycotoxin	N (P)	Range (Min–Max)	Median	Mean	Recovery (%)
AFB_1_	15 (25%)	(0.2–44.9)	1.35	8.7	100
AFB_2_	6 (10%)	(0.1–7.0)	1.7	2.2	81
AFG_1_	n.d	n.d	n.d	n.d	84
AFG_2_	n.d	n.d	n.d	n.d	80.5
OTA	n.d	n.d	n.d	n.d	100
ZEA	n.d	n.d	n.d	n.d	85

Median and mean values were calculated for the contaminated samples.
